# Invasive Snails and an Emerging Infectious Disease: Results from the First National Survey on *Angiostrongylus cantonensis* in China

**DOI:** 10.1371/journal.pntd.0000368

**Published:** 2009-02-10

**Authors:** Shan Lv, Yi Zhang, He-Xiang Liu, Ling Hu, Kun Yang, Peter Steinmann, Zhao Chen, Li-Ying Wang, Jürg Utzinger, Xiao-Nong Zhou

**Affiliations:** 1 National Institute of Parasitic Diseases, Chinese Center for Disease Control and Prevention, Shanghai, People's Republic of China; 2 Department of Public Health and Epidemiology, Swiss Tropical Institute, Basel, Switzerland; 3 Ministry of Health of China, Beijing, People's Republic of China; Biomedical Research Institute, United States of America

## Abstract

**Background:**

Eosinophilic meningitis (angiostrongyliasis) caused by *Angiostrongylus cantonensis* is emerging in mainland China. However, the distribution of *A. cantonensis* and its intermediate host snails, and the role of two invasive snail species in the emergence of angiostrongyliasis, are not well understood.

**Methodology/Principal Findings:**

A national survey pertaining to *A. cantonensis* was carried out using a grid sampling approach (spatial resolution: 40×40 km). One village per grid cell was randomly selected from a 5% random sample of grid cells located in areas where the presence of the intermediate host snail *Pomacea canaliculata* had been predicted based on a degree-day model. Potential intermediate hosts of *A. cantonensis* were collected in the field, restaurants, markets and snail farms, and examined for infection. The infection prevalence among intermediate host snails was estimated, and the prevalence of *A. cantonensis* within *P. canaliculata* was displayed on a map, and predicted for non-sampled locations. It was confirmed that *P. canaliculata* and *Achatina fulica* were the predominant intermediate hosts of *A. cantonensis* in China, and these snails were found to be well established in 11 and six provinces, respectively. Infected snails of either species were found in seven provinces, closely matching the endemic area of *A. cantonensis*. Infected snails were also found in markets and restaurants. Two clusters of *A. cantonensis*–infected *P. canaliculata* were predicted in Fujian and Guangxi provinces.

**Conclusions/Significance:**

The first national survey in China revealed a wide distribution of *A. cantonensis* and two invasive snail species, indicating that a considerable number of people are at risk of angiostrongyliasis. Health education, rigorous food inspection and surveillance are all needed to prevent recurrent angiostrongyliasis outbreaks.

## Author Summary

Eosinophilic meningitis is caused by the rat lungworm (*Angiostrongylus cantonensis*). This parasite is endemic in Southeast Asia, Australia, the Caribbean, and on Pacific Islands. Moreover, the disease is emerging in mainland China, which might be related to the spread of two invasive snail species (*Achatina fulica* and *Pomacea canaliculata*). Thus far, the biggest angiostrongyliasis outbreak in China occurred in 2006 in Beijing, involving 160 patients. However, detailed information about the national distribution of *A. cantonensis* and its intermediate hosts is still lacking, and the importance of the two invasive snail species for disease transmission is not well understood. Therefore, a national survey on the distribution of *A. cantonensis* and its intermediate hosts in China was carried out in 2006/2007. It was found that *A. fulica* and *P. canaliculata* were implicated in most angiostrongyliasis outbreaks, and that the distribution of *A. cantonensis* closely matched that of these snails. The two invasive snail species facilitated the expansion of the parasite, thus probably leading to the emergence of angiostrongyliasis, a previously rare disease, in mainland China.

## Introduction

Eosinophilic meningitis, a potentially fatal disease caused by *Angiostrongylus cantonensis*, is considered an emerging infectious disease in mainland China [1],[2]. The first human case of angiostrongyliasis in mainland China was reported in 1978, and a few more cases were diagnosed until the mid-1990s. Subsequently, several outbreaks have been recorded [1]. The first major angiostrongyliasis outbreak, involving 65 patients, was documented from Wenzhou in Zhejiang province in 1997 [3]. The biggest outbreak in China thus far could be attributed to a freshwater snail, i.e., *Pomacea canaliculata*, and took place in the capital Beijing in 2006 [4]. Of the 160 infected individuals involved in this outbreak, 100 were hospitalized [5]. This outbreak also demonstrated that angiostrongyliasis had moved beyond its traditional endemic areas located in the southeastern coastal regions of China.

The parasite was first described by Chen based on worm specimens collected from pulmonary arteries of rats in Guangzhou (Canton) [6] and Dougherty proposed the name *A. cantonensis* in 1946 [7]. Adult *A. cantonensis* live in the pulmonary arteries of its definitive hosts, i.e., rodents, especially rats, which pass infective first-stage larvae (L_1_) in their feces. The life cycle also involves mollusks, harboring larval stages. In humans, larvae fail to mature, and hence humans and their excreta play no role in the transmission and direct dissemination of the parasite. Humans become infected by ingesting third-stage larvae (L_3_) in raw or undercooked intermediate host mollusks (e.g., snails and slugs) or paratenic hosts (e.g., freshwater prawns, crabs, frogs and fish) [8]–[10]. Lettuce and vegetable juice have also been identified as sources of infection when contaminated with intermediate or paratenic hosts [11],[12]. Due to the low host specificity of *A. cantonensis* it is difficult to control this parasite [1]. Two snail species, i.e., *Achatina fulica* and *P. canaliculata*, are believed to be closely associated with angiostrongyliasis in China. These snails were imported into mainland China in 1931 [13],[14] and 1981 [1],[15], respectively, and have rapidly extended their geographic ranges. Indeed, these two snails are now listed as invasive species by the Chinese government.

In response to the recent angiostrongyliasis outbreak in Beijing that had received considerable national and international attention and mass-media coverage, the Ministry of Health (MoH) of China launched the first national survey on *A. cantonensis*. Here, we report the design and key findings of this survey. Moreover, predictions are made for the spatial distribution of *A. cantonensis* and its intermediate hosts. Finally, recommendations are offered for the prevention of angiostrongyliasis.

## Methods

### Ethics Statement

The project entitled “The first national survey on *Angiostrongylus cantonensis* in China” has been approved by the institutional ethics committee of the National Institute of Parasitic Diseases, Chinese Center for Disease Control and Prevention in Shanghai (ref. no. 2006030101). Animal experiments were carried out in adherence to institutional guidelines for animal husbandry.

### Design of the national survey on *A. cantonensis*

The first national survey pertaining to *A. cantonensis* and its definitive and intermediate hosts in mainland China was implemented in two phases over a 1-year period, i.e., between September and November 2006, and between March and October 2007. Considering that the distribution of *A. cantonensis* is affected by several environmental and ecological factors, the potential distribution of the parasite was first determined. Temperature was selected as the main factor to predict the potential distribution of the parasite and two invasive snail species in China. Since revealing the distribution of both the parasite and two snail species implicated in its transmission was the main aim of this survey, the widest potential distribution of *P. canaliculata*, which has previously been regarded as the most important intermediate host, delineated the survey region. The potential range of *P. canaliculata* in China was predicted using a degree-day model based on temperature data obtained from 149 observing stations across China [16]. A grid with a spatial resolution of 40×40 km was laid over the predicted area, and approximately 5% of the grid cells were randomly selected for sample collection. In each survey grid cell, one village was randomly selected for subsequent field work. The geographic coordinates of the survey villages were recorded using a hand-held global positioning system (GPS) device (GPSmap 70; Kansas, USA).

### Field survey of *A. cantonensis* and its hosts

Rats are the definitive hosts of *A. cantonensis*. Some insectivores also serve as suitable definitive hosts [17],[18]. Therefore, rats (e.g., *Rattus norvegicus*) and insectivores (Soricidae, e.g., *Suncus murinus*) were trapped in fields and in residents' houses. All captured animals were euthanized and dissected to determine the presence of adult *A. cantonensis* in their hearts and lung arteries.

Freshwater snails (e.g., *P. canaliculata* and *Bellamya aeruginosa*), terrestrial snails (e.g., *A. fulica*) and certain terrestrial slugs were collected from the surroundings of the study villages, and from restaurants and markets in the capital town of the counties, and snail farms across the study area, and examined for the presence of *A. cantonensis* larvae. Up to 100 specimens of each species were collected at each study site. The intermediate hosts were artificially digested using routine procedures (incubation in a solution containing 0.2% pepsin and 0.7% hydrochloric acid at 37°C for 2 h) [19]. Additionally, for the examination of *P. canaliculata*, a recently developed method relying on specific lung tissue features of this species was employed [19],[20]. In brief, the lungs were separated from the snail body and opened. The nodules containing *A. cantonensis* larvae were then directly observed under a microscope. Paratenic hosts were also collected from markets and restaurants, and examined for L_3_ using an artificial digestion method.

*A. cantonensis* larvae were identified based on distinct morphological criteria described elsewhere [21]. For quality control purpose, larvae identified as *A. cantonensis* from approximately 10% of the foci where *A. cantonensis* was found to be endemic were intragastrically injected into Sprague-Dawley (SD) rats. The animals were then maintained in the laboratory to confirm the identity of adult worms.

### Analysis

An area was considered *A. cantonensis* endemic if the parasite was detected in any kind of animals captured in the field. The geographic locations of these sampling sites were linked to an existing geographic information system (GIS), using the software ArcGIS version 9.1 (ESRI, USA). Subsequently, ordinary kriging, a statistical technique for spatial prediction [22], was performed, and thus a smoothed risk map of the *A. cantonensis* infection prevalence in *P. canaliculata* was produced.

## Results

### *A. cantonensis* hosts and their infection status

**Figure 1:**
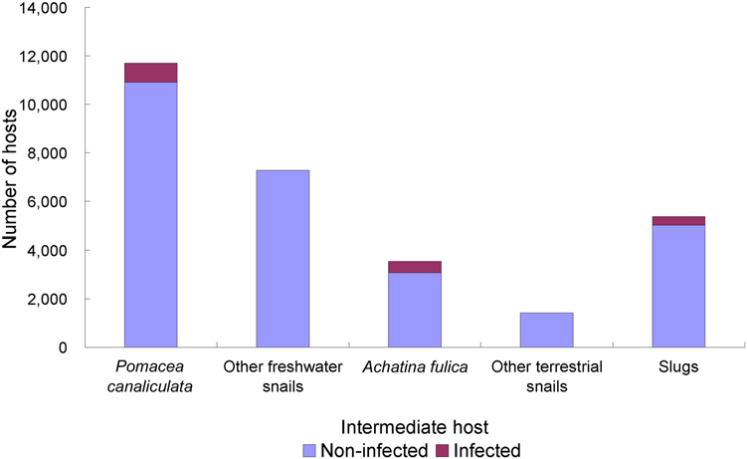
Number and infection status of potential intermediate hosts of *Angiostrongylus cantonensis* examined during the first national survey in mainland China, 2006/2007.

The first national survey pertaining to *A. cantonensis* in China was implemented in 164 counties belonging to 19 provinces. A detailed list of the surveyed locations is available from the corresponding author upon request. Various mollusks were collected, belonging to one of the three following groups: (i) freshwater snails, (ii) terrestrial snails, and (iii) terrestrial slugs. All collected specimens were deposited in the “Preservation Center for Parasite Specimens in China” (http://www.psic.cn), and further details are available from this center upon request. Overall, 11,709 *P. canaliculata* were screened, 6.8% of which harbored L_3_ of *A. cantonensis*. The prevalence among the other freshwater snails (a total of 7,287 specimens were examined) was only 0.05%. Of 3,549 *A. fulica* examined, 13.4% were infected. The infection prevalence among the 1,421 other terrestrial snail specimens was only 0.3%. Finally, 5,370 terrestrial slugs were dissected, revealing an infection prevalence of 6.5%. Hence, the endemicity of *A. cantonensis* in mainland China is primarily attributable to *P. canaliculata*, *A. fulica* and terrestrial slugs (Figure 1).

Of the 711 potential host animals trapped during the field surveys, 32 were found to be infected with *A. cantonensis* (31 *R. norvegicus* and one *R. flavipectus*; overall prevalence: 4.2%). None of the 46 insectivores (*Suncus murinus*) were infected. The 652 paratenic hosts collected during the survey included frogs, shrimps, crabs, toads and fish. No *A. cantonensis* was found in any of these animals.

**Figure 2:**
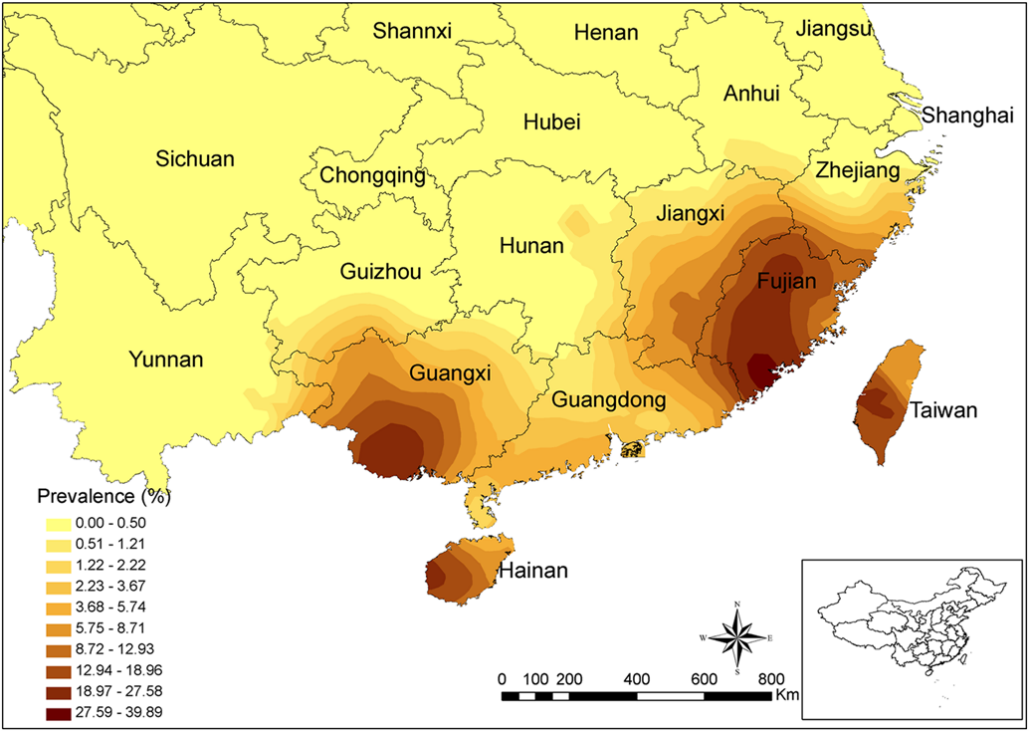
Predicted *Angiostrongylus cantonensis* prevalence within *Pomacea canaliculata* in mainland China, 2006/2007. The map is based on the currently available data regarding the prevalence of *A. cantonensis* within *P. canaliculata*, smoothed by ordinary kriging. The predicted prevalences were then stratified into 10 categories by the smart quantiles technique.

The prediction map of the *A. cantonensis* prevalence in *P. canaliculata* in China, using an ordinary kriging approach with a spherical model, highlighted two potential clusters with prevalences of 19–28% in Guangxi province and 28–40% in Fujian province, respectively (Figure 2).

### Geographic distribution of *A. cantonensis* and its main hosts

**Figure 3:**
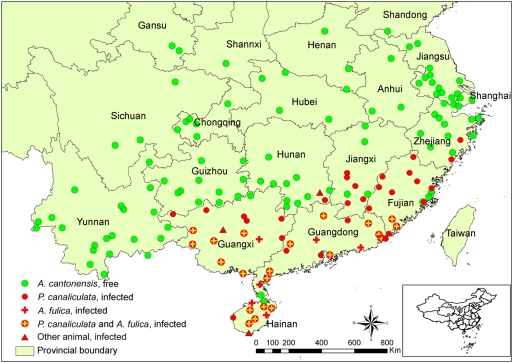
Occurrence of *Angiostrongylus cantonensis* according to hosts in mainland China, 2006/2007. Each point represents a county where the national survey was implemented. All field sites are located within a region delimited by northern latitude 18°13′–34°50′ and eastern longitude 97°50′–122°07′. The results pertaining to two invasive snail species, i.e., *Pomacea canaliculata* and *Achatina fulica*, are highlighted, and the infection status of other animals was omitted whenever infected snails were found in the same places.

Figure 3 shows the current distribution of *A. cantonensis* at county level in China. The parasite was identified in 59 of the 164 surveyed counties (36.0%). Most of the *A. cantonensis*-endemic areas were defined by infections in *P. canaliculata* and/or *A. fulica* snails. Only in three counties infected rats were found, but the presence of the parasite in intermediate hosts could not be ascertained. Seven provinces in southeastern China (i.e., Hainan, Guangdong, Guangxi, Fujian, Jiangxi, Hunan, and Zhejiang) were identified as *A. cantonensis* endemic.

**Figure 4:**
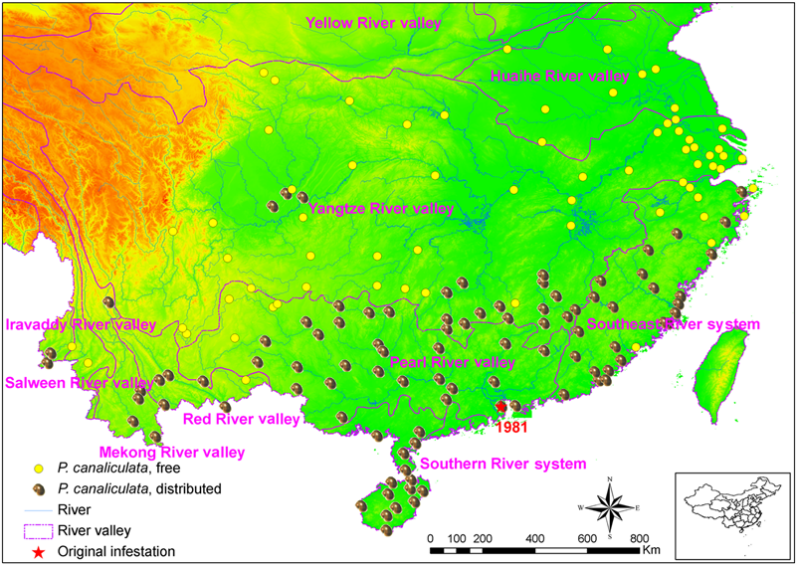
Distribution of *Pomacea canaliculata* in mainland China, 2006/2007. All field sites are located in a region delimited by the northern latitude 18°13′–34°50′ and eastern longitude 97°50′–122°07′. Also shown are the terrain and the waterways along with the point of introduction of *P. canaliculata* (Zhongshan city; red star). The elevation is depicted with green indicating the lowest elevation and red indicating the highest elevations.

*P. canaliculata* was introduced in Zhongshan city, Guangdong province in 1981 [15]. As shown in Figure 4, *P. canaliculata* is now well established in southern China in a band spanning northeast-southwest. A separate endemic area is located in the Sichuan basin. The snail now colonizes almost the entire Pearl River valley, the Southern River system and the Southeast River system. The snail has also been observed in mountainous areas at high elevations in Yunnan province. Moreover, *P. canaliculata* snails have crossed from the Pearl River valley into the Yangtze River valley, and already inhabit the southeast section of the latter.

**Figure 5:**
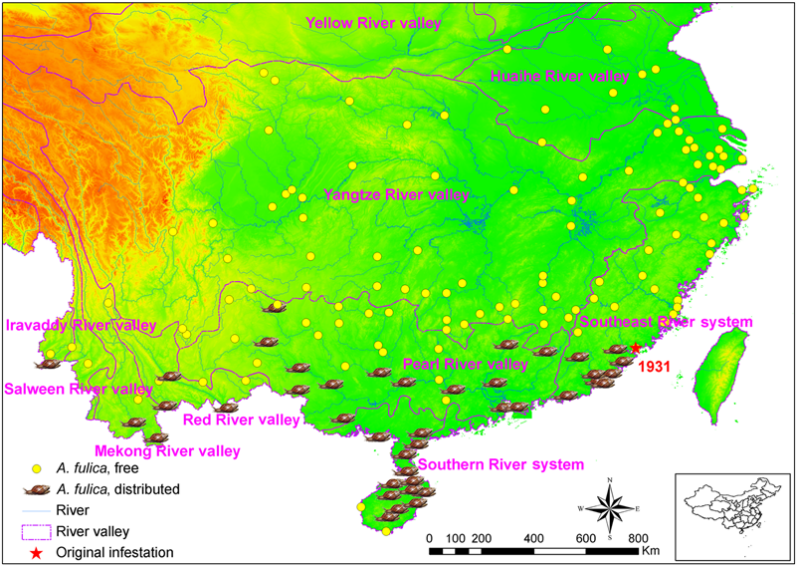
Distribution of *Achatina fulica* in mainland China, 2006/2007. All field sites are located in a region delimited by the northern latitude 18°13′–34°50′ and eastern longitude 97°50′–122°07′. The terrain and the waterways along with the point of introduction of the terrestrial *A. fulica* (Xiamen; red star) are also presented in this map. The elevation is depicted with green indicating the lowest elevation and red indicating the highest elevations.

Figure 5 shows that *A. fulica* has a more focal distribution than *P. canaliculata*, although the former species had been introduced into China half a century earlier than the latter. At present, *A. fulica* is known to occur in the provinces of Guangdong, Hainan, and Guangxi, in the southern areas of Yunnan and Fujian provinces, and in one county of Guizhou province. Unlike *P. canaliculata*, *A. fulica* occurs only south of 25° N latitude, and does not appear to be associated with major river networks.

### Intermediate host snails on markets and in restaurants

*P. canaliculata* snails were found on markets and/or in restaurants in 21 counties, whereas *A. fulica* snails were detected in three counties only. Infected *P. canaliculata* and *A. fulica* were found in nine and two counties, respectively. Additionally, two native freshwater snail species, i.e., *B. aeruginosa* and *Cipangopaludina chinensis*, were commonly found to be on sale in many markets and restaurants. *C. chinensis* is one of the key intermediate hosts of *A. cantonensis* in Taiwan and, in the current survey, infected specimens were detected in one restaurant in Fujian province. To our knowledge, this is the first report of *A. cantonensis*-infected *C. chinensis* from mainland China. On two markets in Guangdong and Guangxi provinces, infected *B. aeruginosa* snails were detected.

### Commercial snail farming

Only two commercial snail farms for *P. canaliculata* (located in Jiangsu and Jiangxi provinces) and one for *A. fulica* (in Zhejiang province) were identified during this survey. None of the snails collected in these farms was infected with *A. cantonensis*.

## Discussion

Eosinophilic meningitis caused by *A. cantonensis* is endemic in Southeast Asia, Australia, the Pacific Islands and the Caribbean. To date, more than 2,800 human cases have been reported [23]. It had been suggested that the parasite was dispersed from East Asia to other regions in two important hosts, i.e., rats (definitive host) and *A. fulica* (intermediate host) especially during World War II [24]. Today, the parasite is still expanding its range and the associated disease is emerging in some regions, particularly China [1], [25]–[28].

The results of the first national survey on the distribution of *A. cantonensis* and its hosts in China reported here indicate that there is a need for strengthening food safety inspections and food-borne disease surveillance. Long-distance trade, biological invasion and animal migration are contributing to the emergence of new diseases and the re-emergence of diseases that have previously been controlled [29]–[32]. Angiostrongyliasis in mainland China is an example of such an emerging food-borne disease. Its spread can be linked to the introduction, farming and consumption of certain snail species. Extrapolating from recent observations, the incidence of angiostrongyliasis is likely to further increase in China, although the 2006 outbreak in Beijing triggered considerable attention and a change in attitudes toward this parasitic infection not only in the medical and research community, but also the general public.

The results of the national survey can be summarized as follows. First, the *A. cantonensis*-endemic area is very wide, covering seven southern provinces. However, not a single snail or rat infected with *A. cantonensis* was found in Yunnan province. This observation comes as a surprise, since the parasite was first documented in Yunnan some 20 years ago [33], and several outbreaks have occurred subsequently [34],[35], most recently in Dali (early 2008). Hence, Yunnan must clearly be considered a potentially endemic province.

Second, several freshwater and terrestrial snail species were found on local markets and in restaurants, and *A. cantonensis*-infected *P. canaliculata* and *A. fulica* clearly destined for human consumption were recorded. This observation suggests that the transmission of *A. cantonensis* to humans is ongoing, and that the health education and awareness raising campaigns initiated after the 2006 outbreak in Beijing – targeting consumers, health personnel and officials alike – must be improved since they appeared to have failed yet to stop the sale and consumption of infected snails. It follows that the impact of the previous health education campaigns through mass media to change human behavior has probably been overestimated, because angiostrongyliasis outbreaks continued in Guangdong province in 2007 [36] and Yunnan province in early-2008, involving six and 41 patients, respectively.

Third, culturally-routed dietary habits of certain ethnic groups increase the risk of *A. cantonensis* infection. For example, the consumption of raw or undercooked freshwater snails is held responsible for the early-2008 angiostrongyliasis outbreak in Dali. As a direct consequence of the booming inland tourism in China, the interest in minority dishes is growing, and ethnic dining has become popular among tourists and wealthy urban residents alike. Travelers to endemic regions with a tradition of preparing snails for human consumption should be better informed about the risks associated with certain dishes, and food inspection and hygiene regulations need to be enforced.

Fourth, among the different factors facilitating the spread and transmission of *A. cantonensis* in China, the two invasive mollusk species *P. canaliculata* and *A. fulica*, play a central role. A range of mollusks can serve as intermediate hosts of *A. cantonensis* and were examined during the national survey. The prevalence of *A. cantonensis* infection among *P. canaliculata*, *A. fulica* and terrestrial slugs was found to govern the endemicity of this parasite in China. However, terrestrial slugs had rarely been found to be associated with human angiostrongyliasis; the only exception being their occasional use in local traditional medicine [37]–[39]. Thus, *P. canaliculata* and *A. fulica* are probably responsible for most angiostrongyliasis cases in China. Both snails not only expand their range, but also frequently go on the table for human consumption.

Interestingly, *P. canaliculata* and *A. fulica* have facilitated the spread of the endemic area of *A. cantonensis* rather than the introduction of a new pathogen. Man-made ecological transformations and climate change are important drivers of the spread of exotic species and their establishment in new areas [29],[40],[41]. The emergence of several infectious diseases has already been attributed to the invasion of efficient vectors or hosts involved in their life cycle [42]. These two invasive snail species impact on the endemicity and transmission of *A. cantonensis* in at least two ways. First, the invasion of these snails facilitates the establishment of the life cycle of the parasite and thus increases the chances for an exposure of native mollusks to *A. cantonensis* in existing endemic areas. Previous experiments indeed documented a superior susceptibility of these snails to *A. cantonensis* compared to native snails [43]. Second, these invasive snails accelerate the spread of *A. cantonensis* since they rapidly expand their range, resulting in the local establishment of the snail and – sometimes – the parasite life cycle in previously non-endemic areas.

*A. fulica* was recorded for the first time in mainland China in 1931 [14]. It has been suggested that eggs of *A. fulica* were accidentally imported from Singapore with shipments of plants, and that an initial snail population became established in Xiamen (Amoy) [13]. These terrestrial snails are nocturnal and become active under high-humidity conditions [44]. The snails feed on plants and deposit their eggs in the soil nearby. This behavior facilitates their dispersal through long-distance transportation of pot plants [13]. Since their unintentional introduction, *A. fulica* spreads across southern China, probably facilitated by the rapid expansion of long-distance trade and an increasing demand for farmed plants going hand-in-hand with China's ongoing economic development. It has even been speculated that *A. fulica* invaded China more than once. For example, the snail populations in Yunnan province might derive from trade with Indochina (Mekong basin) rather than eastern China [45]. The public health significance of *A. fulica* in mainland China was only noted when a parasitologically-confirmed case of angiostrongyliasis found in 1984 could be linked to this snail [46]. However, the consumption of *A. fulica* snails is generally less popular than that of *P. canaliculata* in mainland China.

The freshwater snail *P. canaliculata* was deliberately introduced into China for human consumption. The invasion process can be stratified into three stages, i.e., (i) introduction, (ii) establishment, and (iii) spread [47]. It was first imported into Zhongshan city in Guangdong province approximately 30 years ago [1]. Subsequently, the snail was farmed in most southern provinces with commercial aims [15]. However, within a few years, the snail also became established outside due to abandoning of farms and deliberate release [15]. Currently, the snails have reached 30° N latitude and had been found as high as 1,960 m above sea level in Yunnan province. It is conceivable that the dense river networks in eastern and southern China contributed to the dispersal of this snail. The isolated snail population in the Sichuan basin has expanded freely in this area for about 20 years. The easternmost natural colonies were observed in Zhoushan in Zhejiang province, suggesting a line from Zhoushan to the Sichuan basin south of which climate conditions are suitable for the snails to thrive. This line might move further northward as a consequence of global warming [48]. The public health significance of *P. canaliculata* was emphasized by the first major angiostrongyliasis outbreak in Wenzhou in 1997 [3]. The results of the national survey presented here suggest a close relationship between the endemicity of *A. cantonensis* and the area where *P. canaliculata* breed two or even three generations per year [16], suggesting that *A. cantonensis* largely depends on this freshwater snail for its expansion in China. Although *P. canaliculata* in the whole endemic area of *A. cantonensis* were found to be infected, point prevalences of infection are heterogeneous: two heavily endemic areas could be identified in the provinces of Fujian and Guangxi, respectively. The snail is indeed responsible for many sporadic cases recorded throughout Fujian province. However, it remains to be investigated why no angiostrongyliasis cases have been observed thus far in Guangxi province.

Although both *A. fulica* and *P. canaliculata* appear to have contributed to the emergence of angiostrongyliasis in China, several characteristics of *P. canaliculata* suggest that this species is mainly responsible for the spread of *A. cantonensis*. This claim is supported as follows. First, the aquatic *P. canaliculata* probably spread along waterways, accelerated through flooding events. This might partly explain why *P. canaliculata* more rapidly expanded its range than *A. fulica*, which appears to largely depend on human-facilitated transport. Second, the area colonized by *P. canaliculata* also expands far beyond that of *A. fulica* despite a considerably longer presence of the latter in China. Third, the consumption of *P. canaliculata* is more popular than that of *A. fulica*. During the national survey, for example, *P. canaliculata* was on sale in 21 counties, while *A. fulica* was only noted in three counties.

The national survey shed light on different important aspects regarding the distribution of *A. cantonensis* and its hosts in China. The results indicate a need for more pointed attention to this emerging threat through awareness-raising campaigns among the medical community, the establishment of a hospital-based sentinel surveillance system, improved community-based health education and strengthening of food safety inspection. A number of pressing research questions could also be identified. For example, the model for predicting the prevalence of *A. cantonensis* within *P. canaliculata* identified two high-prevalence clusters. However, the accuracy of this prediction has not been assessed since no ground truthing of the predictions has been made thus far. The small-scale distribution, the range of hosts and the host-parasite compatibility should also be investigated to deepen our understanding of the transmission dynamics.

In conclusion, the first national survey revealed the distribution of *A. cantonensis* and two invasive snail species, i.e., *P. canaliculata* and *A. fulica*, and the pivotal role of these invasive snails for the transmission of this parasite. The results of the survey also suggest that people are at risk of angiostrongyliasis through consumption of raw or undercooked snails infected with *A. cantonensis* that are found in many markets and restaurants. Continued health education, rigorous food inspection, and hospital-based surveillance are needed to prevent recurrent angiostrongyliasis outbreaks in China.
